# Machine Learning-Based Mortality Prediction Model for Critically Ill Cancer Patients Admitted to the Intensive Care Unit (CanICU)

**DOI:** 10.3390/cancers15030569

**Published:** 2023-01-17

**Authors:** Ryoung-Eun Ko, Jaehyeong Cho, Min-Kyue Shin, Sung Woo Oh, Yeonchan Seong, Jeongseok Jeon, Kyeongman Jeon, Soonmyung Paik, Joon Seok Lim, Sang Joon Shin, Joong Bae Ahn, Jong Hyuck Park, Seng Chan You, Han Sang Kim

**Affiliations:** 1 Department of Critical Care Medicine, Samsung Medical Center, Sungkyunkwan University School of Medicine, Seoul 06351, Republic of Korea; 2 Department of Biomedical Systems Informatics, Yonsei University College of Medicine, Seoul 03722, Republic of Korea; 3 Institute for Innovation in Digital Healthcare (IIDH), Severance Hospital, Seoul 03722, Republic of Korea; 4 Yonsei University College of Medicine, Seoul 03722, Republic of Korea; 5 KB Kookmin Bank, Seoul 04534, Republic of Korea; 6 Division of Pulmonary and Critical Care Medicine, Department of Medicine, Samsung Medical Center, Sungkyunkwan University School of Medicine, Seoul 06351, Republic of Korea; 7 Theragen Bio, Seongnam-si 13488, Republic of Korea; 8 Department of Radiology, Yonsei University College of Medicine, Seoul 03722, Republic of Korea; 9 Yonsei Cancer Center, Division of Medical Oncology, Department of Internal Medicine, Yonsei University College of Medicine, Seoul 03722, Republic of Korea; 10 KAKAO Brain, Seongnam-si 13529, Republic of Korea; 11 Graduate School of Medical Science, Brain Korea 21 Project, Yonsei University College of Medicine, Seoul 03722, Republic of Korea

**Keywords:** artificial intelligence, prognosis prediction, clinical decision support system, critically ill cancer patients

## Abstract

**Simple Summary:**

This study describes a new machine-learning-based 28-day mortality prediction model in adult cancer patients admitted to the intensive care unit (ICU). A total of 6900 patients in three patient cohorts were used for the development, internal validation, and external validation, respectively, leading to the generation of a reliable model with high sensitivity and specificity. The CanICU uses nine variables that can be easily obtained in a practical ICU, with the potential benefit of critical care and avoiding unnecessary suffering. Furthermore, this is the largest patient cohort for developing a cancer patient-specific model. CanICU offers improved performance for predicting short- and long-term mortality in critically ill cancer patients admitted to the ICU. CanICU can help physicians determine how to allocate ICU care for patients with cancer according to objective mortality risk.

**Abstract:**

Background: Although cancer patients are increasingly admitted to the intensive care unit (ICU) for cancer- or treatment-related complications, improved mortality prediction remains a big challenge. This study describes a new ML-based mortality prediction model for critically ill cancer patients admitted to ICU. Patients and Methods: We developed CanICU, a machine learning-based 28-day mortality prediction model for adult cancer patients admitted to ICU from Medical Information Mart for Intensive Care (MIMIC) database in the USA (*n =* 766), Yonsei Cancer Center (YCC, *n =* 3571), and Samsung Medical Center in Korea (SMC, *n =* 2563) from 2 January 2008 to 31 December 2017. The accuracy of CanICU was measured using sensitivity, specificity, and area under the receiver operating curve (AUROC). Results: A total of 6900 patients were included, with a 28-day mortality of 10.2%/12.7%/36.6% and a 1-year mortality of 30.0%/36.6%/58.5% in the YCC, SMC, and MIMIC-III cohort. Nine clinical and laboratory factors were used to construct the classifier using a random forest machine-learning algorithm. CanICU had 96% sensitivity/73% specificity with the area under the receiver operating characteristic (AUROC) of 0.94 for 28-day, showing better performance than current prognostic models, including the Acute Physiology and Chronic Health Evaluation (APACHE) or Sequential Organ Failure Assessment (SOFA) score. Application of CanICU in two external data sets across the countries yielded 79–89% sensitivity, 58–59% specificity, and 0.75–0.78 AUROC for 28-day mortality. The CanICU score was also correlated with one-year mortality with 88–93% specificity. Conclusion: CanICU offers improved performance for predicting mortality in critically ill cancer patients admitted to ICU. A user-friendly online implementation is available and should be valuable for better mortality risk stratification to allocate ICU care for cancer patients.

## 1. Introduction

Recent advances in anticancer therapeutics and supportive care have led to improved survival in patients with cancer [1]. Active anticancer treatment and prolonged patient survival increase the risk of either treatment-related or cancer-specific complications. As a result, the admission of critically ill cancer patients to the intensive care unit (ICU) is increasing, accounting for up to 15% of all ICU patients [2,3,4]. Until recently, admission of patients with cancer to the ICU was often discouraged because of the risk of an unfavorable outcome, patient refusal, and unreliable triage criteria for ICU admission [5,6,7]. While critical care has improved survival rates in cancer patients over the decades, it is still challenging to identify who may benefit from intensive care [8,9,10].

The decision-making process of whether to or not to admit a critically ill cancer patient to the ICU is based on complex considerations, such as age, tumour burden, comorbidities or life expectancy, the identification of risk factors for poor prognosis, number of organ failures, and the need for mechanical ventilation [11,12,13,14]. Although prognostic models for critically ill cancer patients were developed, these models are not externally validated nor publicly available [15,16,17]. There is an unmet clinical need for improved short- and long-term prognostic tools for guiding critically ill cancer patients to increase the potential benefit of critical care and avoid unnecessary suffering.

Machine learning (ML) is the creation of systems that can learn from and anticipate data without being explicitly programmed. It is especially beneficial in situations where signals and data are created faster than the human brain can analyse them [18]. Most ICU prognostic models are based on logistic regression [19,20]. While the logistic regression model requires the statistical assumption of the independent and linear relationship between outcome and exploratory variables, the advantage of the ML approach includes the unbiased analysis of a large number of covariates, integration of nonlinear associations, and interaction terms [21,22]. Recently, advanced ML-based modeling has shown promising results for predicting patient survival for those admitted to the ICU [23,24,25,26].

This study describes a new ML-based mortality prediction model for critically ill cancer patients admitted to the ICU. The program, called CanICU, harnesses electronic patient records during the first 24 h before the ICU admission to predict short-term and long-term mortality.

## 2. Materials and Methods

### 2.1. Patients

We conducted a retrospective study of all critically ill cancer patients admitted to the ICUs at Yonsei Cancer Center (YCC) (From 1 January 2008 to 31 December 2017, a 2615-bed, university affiliated tertiary referral hospital in Seoul, Republic of Korea). The YCC cohort was divided into a derivation cohort (70%) for developing a preliminary model of CanICU and an internal validation cohort (30%). We identified patients aged 18 years or older with a diagnosis of cancer (ICD-10:C00-C99 as the primary diagnosis) who stayed in the ICU for more than 24 h. Only the first admission was selected for analysis for patients who had multiple ICU admissions during the study period.

For the validation cohort, critically ill cancer patients were used, admitted to the ICUs of the Samsung Medical Center (SMC) (a 1989-bed, university affiliated tertiary referral hospital in Seoul, Republic of Korea) from 1 January 2011 to 31 December 2017 and the cancer patients in the Medical Information Mart for Intensive Care (MIMIC)-III (version 1.4). The institutional review boards of all participating hospitals approved this study and waived the requirement for informed consent because of the observational nature of the research. All patient records and data were anonymized and de-identified before analysis.

### 2.2. Source of Data

This study made use of data from the YCC Clinical Data Retrieval System, the SMC Clinical Data Warehouse Darwin-C database, and the MIMIC-III database. The SMC data set and MIMIC-III dataset were used for the validation cohort, while the YCC dataset was used for the derivation and validation cohorts. MIMIC-III is a clinical database of over 38,000 ICU patients (medical, surgical, coronary care, and neonatal) admitted to Beth Israel Deaconess Medical Center (Boston, MA, USA) from June 2001 to October 2012 [27]. Our access to the database was approved by its administrators. The study was approved by the Institutional Review Board (IRB) of Severance Hospital (YCC, IRB approval number, 4-2018-0940) and by the Institutional Review Board of Samsung Medical Center (SMC, IRB approval number, 2018-11-091).

### 2.3. Outcomes

The primary outcome of the study was the prediction of 28-day mortality after ICU admission. The model trained to predict the primary outcome was also validated for the secondary outcome: 1-year mortality. Outcomes were compared according to primary cancer type.

### 2.4. Predictor Variables

**Figure 1:**
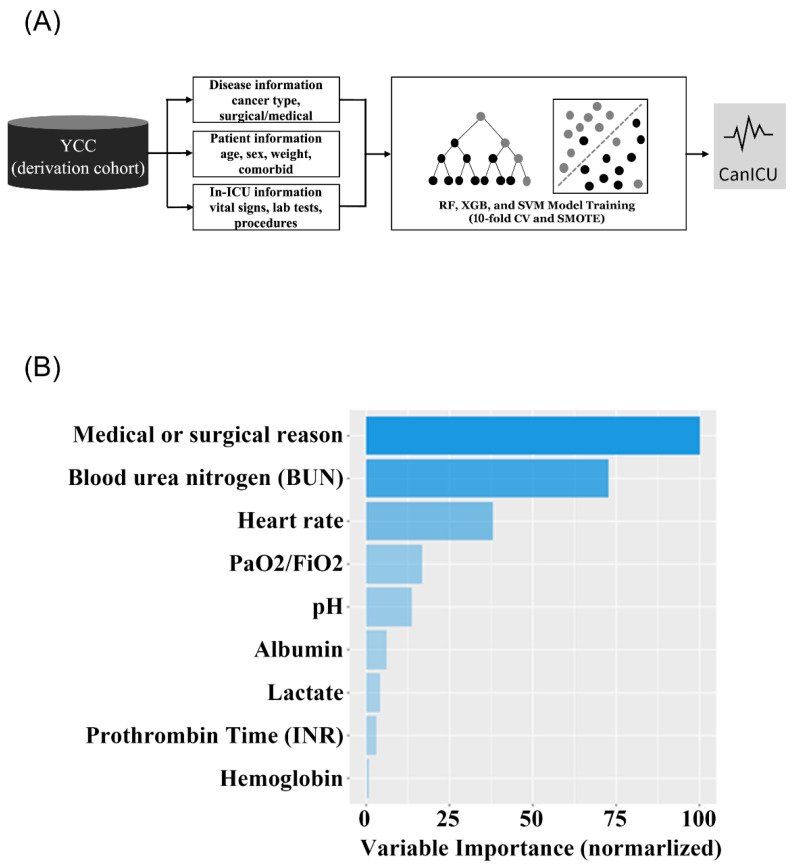
Architectural overview and feature importance of CanICU: (**A**) Architectural overview of CanICU. (**B**) Feature importance for predicting 28-day mortality. YCC, Yonsei Cancer Center; RF, random forest; XGB, xgboost; SVM, support vector machine; CV, cross validation; SMOTE, synthetic minority oversampling technique. Evaluation of short term 28-Day mortality prediction in CanICU.

Predictors were selected among 83 variables in the YCC cohort a priori based on our clinical experience and previous literature describing risk factors for the mortality of critically ill cancer patients (Figure 1A, Table S1) [14,28,29,30,31]. Disease-related information (primary cancer type, admission lesion), patient information (age, gender, body weight, comorbidities, and in-ICU information (vital signs, laboratory variables, intervention) were included. Moreover, we used a generic method, using r > 0.7 in at least one correlation as the criteria for collinearity. The logistic model assumption (i.e., no interaction factors and a linear relationship between the logit and continuous variables) was confirmed. A logistic regression analysis utilizing a forward stepwise inclusion approach, with a *p*-value of 0.05 as the starting point. Then, recursive feature elimination was applied within the YCC cohort (Figure 1A). Cancer was defined according to the International Classification of Diseases, Tenth Revision (ICD-10) codes from prior admissions in the YCC and SMC cohorts. In the MIMIC-III data set, International Classification of Diseases, Ninth Revision (ICD-9) codes were used. See Table S2 for details of codes used to identify each cancer patient in ICD-9 and ICD-10. For each predictor variable, the measured value within 24 h after ICU admission was used. Laboratory variables and vital signs use the most harmful values for 28-day mortality risk captured within 24 h prior to admission to the ICU (see Table S1 for definitions of whether laboratory tests and vital signs are harmful). A matrix containing the correlation coefficient between independent variables was used to test for collinearity.

### 2.5. Modeling Strategies

To develop a prediction model, the patient from the YCC cohort was divided into derivation (70%) and internal validation (30%) cohorts. We compared three modeling strategies with Sequential Organ Failure Assessment (SOFA) scores: (1) random forest, (2) extreme gradient boosting (XGBoost), (3) support vector machine (SVM). In the derivation cohort, we oversampled the minority class (fatal cases within 28 days) five times using the synthetic minority oversampling technique (SMOTE) to balance classes with and without the primary outcome [32]. The hyperparameters of models were defined through 10-fold cross-validation. We report the result from the random forest for brevity, which showed the most robust sensitivity in the three datasets in the manuscript.

The random forest is a decision tree-based ensembling machine learning model that reduces variance by aggregating many decision trees. Each tree in the random forest is developed based on a random sample drawn by bootstrapping from the original training set. At each bootstrap sample of the original training data, classification and regression (CART) trees are generated using only a given number of randomly chosen variables to determine the split [33]. CARTs are binary decision trees built by repeatedly splitting the data in a node into child nodes from the root, which holds the entire sampled data. The final prediction is determined by the majority of the votes from the multiple trees. The optimal number of variables to determine splitting at each tree node was defined by 4 through cross-validation. The model developed in this method was named ‘CanICU’ (Figure 1A). Only patients with all selected nine variables were included for the model development and validation.

### 2.6. Accuracy Comparisons

Predicted probabilities were calculated for 28-day mortality in the validation cohorts using the optimally tuned random forest model from the derivation cohort. The following metrics were used to evaluate the performance of the ‘CanICU’ model: area under the receiver operating curve (AUC), Cohen’s Kappa statistic, and F1 score. In addition, sensitivity, specificity, positive predicted value (PPV), negative predicted value (NPV), and Brier score were used as model discrimination indices. The performance of ‘CanICU’ was compared with the previously published SOFA score and Acute Physiology and Chronic Health Evaluation (APACHE)-III score in validation cohorts (internal validation, SMC (SOFA score only), and MIMIC-III cohorts) [34]. To inspect the clinical validity of the derived model, the Gini importance score was visualized [35]. All analyses were performed using R version 3.5.0 (The R Foundation for Statistical Computing). A *p* value < 0.05 denoted statistical significance. All hyperparameters and source codes for this work are available at https://github.com/dr-you-group/CanICU, accessed on 16 January 2023.

## 3. Results

### 3.1. Development of CanICU

A total of 6900 patients were included from three independent cohorts. From 2008 to 2017, a total of 3571 critically ill cancer patients in the YCC cohort were analysed with 83 clinical or laboratory variables as a discovery cohort (Figure S1). As a validation, the MIMIC-III database in the USA (*n* = 766) and Samsung Medical Center in Korea (SMC, *n* = 2563) were used. All patients had active cancer disease. Cancer survivors without cancer recurrence were excluded.

The baseline demographics are shown in Table 1 compared with the SMC cohort and MIMIC-III data as validation cohorts. The overall 28-day mortality rate was 14.0% (968 out of 6900 patients from three cohorts), with an overall 1-year mortality rate of 35.6% (2458 out of 6900 patients). Twenty-eight-day mortality was 10.2%/12.7%/36.6%, and 1-year mortality was 30.0%/36.6%/58.5% in the YCC, SMC, and MIMIC-III cohorts, respectively (Table 1). In the YCC cohort, the median age was 64 years, and 2383 (66.7%) patients were male. The most common primary diagnosed site of cancer was the liver (820 patients, 23.0%), followed by colorectal cancer (693 patients, 19.4%). Of the 3571 patients, 2976 (83.3%) patients were admitted for post-operation management. The median SOFA score for the initial 24 h period at ICU admission was 6 (range, 2–10; Table 1).

Next, we developed machine learning-based ‘CanICU’ (https://yonsei-cancer-center.shinyapps.io/CanICU, accessed on 16 January 2023) using the YCC discovery cohort (*n =* 2499; 70% of the YCC cohort). A total of nine clinical and laboratory features were selected in the final prediction model based on a random forest algorithm (Figure 1B). These predictive features include the primary reason for ICU admission (medical reason (i.e., sepsis) vs. surgical problem (i.e., postoperative care)), blood urea nitrogen (BUN), heart rate, PaO_2_/FiO_2_ ratio, pH, albumin, lactate, prothrombin time (PT), hemoglobin in CanICU.

**Figure 2:**
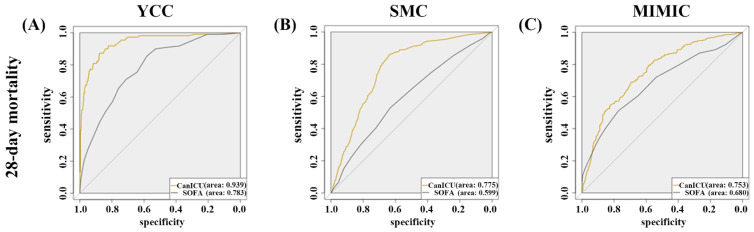
Receiver operating characteristic curves of the CanICU model and SOFA score for predicting 28-day mortality and one-year mortality: (**A**) YCC model for 28-day mortality. (**B**) SMC model for 28-day mortality. (**C**) MIMIC-III model for 28-day mortality. YCC, Yonsei Cancer Center; SMC, Samsung Medical Center; MIMIC, Medical Information Mart for intensive care.

The overall sensitivity, specificity, positive predictive value, negative predictive value, and area under the receiver operating curve (AUC) was compared to analyse the prediction accuracy of CanICU with conventional scores (SOFA and APACHE-III) in the internal YCC (*n =* 1072; 30% of the YCC cohort), SMC validation cohort (*n =* 2563), and MIMIC-III validation cohort (*n =* 766) (Figure 2, Table 2, and Figure S2). CanICU yielded a sensitivity (true positive rate) of 95.5% and specificity (true negative rate) of 72.9% to predict 28-day mortality, showing better performance, compared to the SOFA score (sensitivity, 85.5%; specificity, 58.2%) in the YCC validation cohort (Table 2). Likewise, 88.9% sensitivity/57.5% specificity in the SMC cohort and 78.6% sensitivity/58.8% specificity in the MIMIC-III cohort were observed (Table 2). CanICU showed a high negative predictive value (NPV) of 99.3% in the YCC cohort, suggesting that CanICU predicts cancer patients who benefit from intensive care.

The derived model predicted short-term mortality better than the SOFA score in the internal validation cohort (Figure 2 and Figure S2). The ‘CanICU’ had a better AUC score when predicting the 28-day mortality patients (AUC, 0.939; 95% CI 0.914–0.964) compared with SOFA (AUC, 0.783; 95% CI, 0.741–0.825) in the YCC test dataset (Table 2). Our model was further validated with two external validation cohorts (Table 2). External validation of the SMC cohort (*n =* 2563) and MIMIC-III cohort (*n =* 766) confirmed robustness of CanICU (SMC: AUC of CanICU vs. SOFA, 0.775 (95% CI, 0.751–0.799) vs. 0.599 (95% CI, 0.566–0.632), MIMIC-III: AUC of CanICU vs. SOFA, 0.753 (95% CI, 0.718–0.788) vs. 0.680 (95% CI, 0.639–0.720)). Further, CanICU had a higher AUC compared to the APACHE-III prognostic score in the YCC internal validation cohort (AUC, 0.939 with CanICU vs. 0.784 with the APACHE-III) and MIMIC-III cohort (AUC, 0.753 with CanICU vs. 0.718 with the APACHE-III) (Figure S2). CanICU yielded a higher sensitivity (true positive rate) of 95.5% and a comparable specificity (true negative rate) of 72.9% to predict 28-day mortality, compared to the APACHE-III score (sensitivity, 67.9%; specificity, 80.8%) in the YCC validation cohort (Table S3). Likewise, the APACHE-III score provided 62.5% sensitivity/71.0% specificity in the MIMIC-III cohort. Overall, the results demonstrate that machine learning-based classifiers have a higher classification power than conventional single-value-based approaches.

### 3.2. The Performance of CanICU in Terms of One-Year Mortality

Beyond short-term 28-day mortality prediction, the ‘CanICU’ revealed a better correlation with one-year mortality (AUC, 0.785; 95% CI 0.755–0.816) compared with SOFA (AUC, 0.664; 95% CI, 0.629–0.698) in the internal YCC test dataset (*n =* 1072; 30% of the YCC cohort; Table S4). Further, external validation on the SMC cohort and MIMIC-III cohort confirmed this observation (SMC: AUC of CanICU vs. SOFA, 0.733 (95% CI, 0.712–0.753) vs. 0.538 (95% CI, 0.515–0.561), MIMIC-III: AUC of CanICU vs. SOFA, 0.711 (95% CI, 0.674–0.747) vs. 0.633 (95% CI, 0.594–0.672)). Of note, CanICU showed high specificity (true negative rate, 92.5%/89.0%/88.1% in YCC, SMC, and MIMIC-III cohorts, respectively) compared to the specificity of the SOFA scores (57.3%/51.0%/79.9% in YCC, SMC, and MIMIC-III cohorts, respectively).

**Figure 3:**
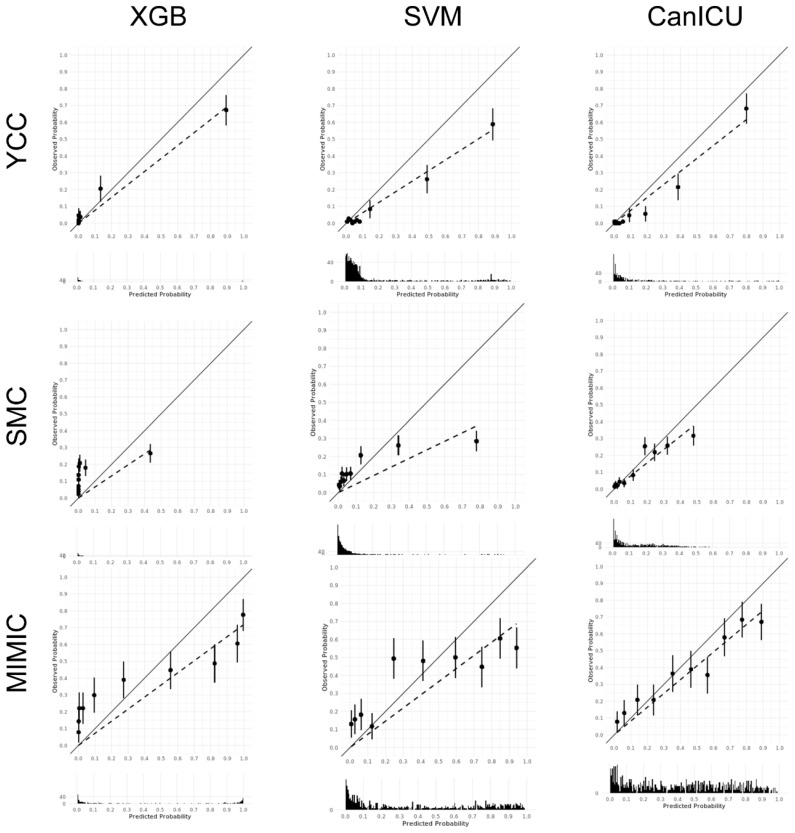
Calibration plots of the preliminary models and CanICU model for predicting 28-days mortality: XGB, xgboost; SVM, support vector machine; YCC, Yonsei Cancer Center; SMC, Samsung Medical Center; MIMIC, Medical Information Mart for Intensive Care.

Overall, the ROC curves of three machine learning models and SOFA score were depicted in Figure S3. Machine learning algorithms, such as extreme gradient boosting (XGBoost), the support vector machine (SVM), or the random forest algorithm, showed better performance than SOFA scores when evaluated by ROC curves (Figure S3 and Table S2). The calibration plots of three machine learning algorithms, including the CanICU (random forest), showed better agreement between prediction and mortality probability in the CanICU, compared to the other two models (Figure 3). Figure S4 shows the precision–recall curve plots for 28-day mortality and 1-year mortality predicted by the CanICU, SOFA score, and APACHE-III score (except for the SMC cohort).

### 3.3. Risk Stratification of the Mortality Using CanICU

**Figure 4:**
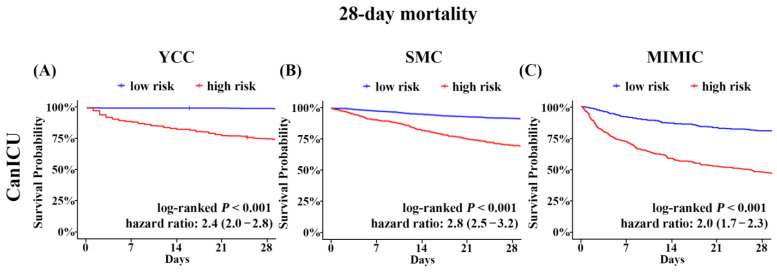
Kaplan-Meier curves according to predicted mortality by the CanICU model in each cohort. (**A**) 28-day survival probability of YCC validation cohort (*n* = 1072). (**B**) 28-day survival probability of SMC cohort (*n* = 2563). (**C**) 28-day survival probability of MIMIC-III cohort (*n* = 766). YCC, Yonsei Cancer Center; SMC, Samsung Medical Center; MIMIC, Medical Information Mart for Intensive Care.

To date, the limited performance of ICU admission criteria for predicting outcomes in cancer patients leads to either the deprivation of the opportunity for intensive care or unnecessary ICU admission in cancer patients. We evaluated the validity of CanICU for the risk stratification of patients admitted to the ICU in cancer patients (Figure 4). We defined the cutoff value for mortality risk, which showed the best sensitivity and specificity ROC curve according to Youden’s index. The survival of identified high- and low-risk patients was analysed using the log-rank test and Cox hazard model. The Kaplan–Meier estimates of the rates of 28-day mortality in the low-risk and high-risk groups were 0.6% and 24.8% in the YCC internal validation cohort, respectively (log-ranked *p* < 0.001; Figure 3). Further, the 28-day mortality rate in the low-risk group was significantly lower than that in the high-risk group in the two external validation cohorts (SMC, 2.9% vs. 23.4% with the high-risk group, log-ranked *p* < 0.001; MIMIC-III, 19.4% vs. 51.7% with the high-risk group, log-ranked *p* < 0.001). The results of the survival analysis for 1-year mortality are reported in Figure S5 (all *p* < 0.001). All calibration plots displayed good concordance between CanICU and observation (Figure 4), suggesting that CanICU has a predictive ability for the likelihood of 28-day mortality in cancer patients. Table S5 shows the performance of CanICU at specific sensitivities in the internal validation cohort (*n* = 1072; 30% of the YCC cohort) and external validation cohorts (SMC and MIMIC-III), respectively.

## 4. Discussion

This study demonstrated that “CanICU,” which employs a machine learning algorithm with electronic medical record (EMR) data, can predict the 28-day mortality of critically ill cancer patients. The CanICU uses nine variables, which can be easily obtained in the emergency room or general ward. In addition to our internal validation, we externally validated our findings using two additional hospitals: one from South Korea and the other from the USA.

Cancer patients are more severely ill than patients without cancer, resulting in poorer short-term and long-term mortality outcomes. Developing a prognostic model for predicting critically ill cancer patients’ outcomes is complex and challenging, involving the several unique characteristics of cancer patients. First, cancer patients have short-term organ failure as a result of cancer therapy, and the consequences are typically transient. Individual reactions to organ dysfunction are also difficult to anticipate since they may be influenced by cancer type, past organ insult status, degree of immunological compromise, and previous general health conditions [36]. Second, long-term persistent antigen exposure and the development of antibiotic resistance impact patients’ chronic immune suppression response to therapies for sepsis and coagulopathy, features that are difficult to include in acuity rating systems. Third, oncologic crises such as tumour lysis syndrome or disseminated intravascular coagulation have a significant mortality risk while probably reflecting tumour destruction. Fourth, many tumour effects and treatment approaches are short-term, with just a narrow window of opportunity to properly treat cancer. Tumour- and therapy-related factors may not indicate mortality but rather the combined impact of tumour growth, destruction, or unfavorable treatment effects. Therefore, the selection variables chosen for the prediction model for critically ill cancer patients are important.

As ICU physicians are flooded with ever-increasing and frequently collected data, ML will become essential to research and clinical practice. ML provides a robust set of tools for describing relationships between features and the outcomes, particularly when they are nonlinear and complex. Furthermore, ML is useful when there are many variables and overcomes the overfitting problems that traditional statistical methods experience. Previous studies have developed mortality prediction models using ML for critically ill patients [23,37,38,39]. In most studies, modeling and validation were performed using only the MIMIC-III data, or modeling was performed using single institution EMR data. Then external validation was performed using MIMIC-III data. The strength of our study is to model via EMR data and validate that model into different trait cohorts and MIMIC-III data. Therefore, our results represent the actual and reliable performance of ‘CanICU’ for other medical institutions.

Futile ICU care is not uncommon [40], which leads to ethical problem, substantial costs, and the burnout of healthcare workers [41]. An accessible, user-friendly web implementation of our scoring procedure has been made available (https://yonsei-cancer-center.shinyapps.io/CanICU, accessed on 16 January 2023). This implementation allows clinicians to use ‘CanICU’ in their practice, such as, for example, as an aid in determining ICU admission allocation of critically ill cancer patients.

Cancer patients and their caregivers usually refuse potentially unnecessary suffering with a low chance of recovery. Given that recent advances in anticancer therapeutic care have led to the improved survival in patients with cancer, CanICU offers a better stratification of cancer patients with a high chance of survival. CanICU had high sensitivity (true positive rate, 95.5%/88.9%/78.6% in YCC/SMC/MIMIC-III cohorts) and a high negative predictive value (99.3%/97.3%/82.7%), suggesting that CanICU help to identify cancer patients who may benefit from intensive care. The 28-day mortality of low-risk patients was 0.6%/2.9%/19.5% in YCC/SMC/MIMIC-III cohorts (Figure 3). Given that high-risk patients had a 28-day mortality of 51.7% in MIMIC-III cohorts, the identification of low-risk patients can encourage cancer patients for full code management.

There are several potential limitations to our study that should be acknowledged. First, ‘CanICU’ was developed using patient data from a single institution, and our results may not be generalizable to other settings. However, we performed external validation with another hospital cohort and MIMIC-III data, and ‘CanICU’ continued to perform well. Second, the data were analysed retrospectively using EMR data not initially designed for the analyses. However, this authenticates our analysis; it confirms its utility in a real-world clinical setting. Third, cancer-specific variables are not included in the nine variables of ‘CanICU’. However, a prospective multicenter cohort study found that the mortality of patients with cancer requiring intensive care mainly depends on the severity of organ failures rather than cancer-related characteristics [42]. Fourth, because the training model did not include critically ill cancer patients who were not admitted to the ICU, potential bias in patient triage may exist in real-world situations. Fifth, we used a complete case analysis and removed patients with missing measurements, which may cause generalizability issues in our study. Sixth, potential ethical issues also need to be considered for the availability of CanICU. To prevent the misclassification of potentially curable patients from not being admitted to the ICU, a discussion with patients and their caregivers also needs to take place. Finally, selection bias might be present as we selected variables available in all three cohorts and the retrospective design of this study. However, the purpose of this study was to develop an easily available model using simple variables and selected variables that could be used commonly in all cohorts.

## 5. Conclusions

In summary, we developed a novel machine learning mortality prediction model, ‘CanICU’, for critically ill cancer patients. Our algorithm, which includes patient demographics, vital signs, and laboratories, can be used to predict the 28-day mortality of critically ill cancer patients and be a good adviser for physicians and patients.

## Figures and Tables

**Table 1:** Baseline characteristics and outcomes.

Variables	YCC (2008–2017) (*n =* 3571)		SMC (2011–2017) (*n =* 2563)		MIMIC (2001–2012) (*n =* 766)		*p* Value
Age, years	64	(55–72)	63	(54–72)	66	(56–75)	<0.001
Sex, male	2383	(66.7)	1668	(65.1)	474	(61.9)	0.030
Primary cancer							
Liver	820	(23.0)	470	(18.3)	107	(14.0)	<0.001
Colorectal	693	(19.4)	358	(14.0)	41	(5.4)	<0.001
Lung	124	(3.5)	149	(5.8)	92	(12.0)	<0.001
Stomach	414	(11.6)	428	(16.7)	15	(2.0)	<0.001
Hematologic malignancy	99	(2.8)	409	(16.0)	170	(22.2)	<0.001
Others *	1421	(39.8)	749	(29.2)	341	(44.5)	<0.001
Admission type							
Medical	595	(16.7)	898	(35.0)	448	(58.5)	<0.001
Surgical	2976	(83.3)	1665	(65.0)	318	(41.5)	<0.001
SOFA at ICU admission	6	(2–10)	6	(4–8)	6	(4–8)	<0.001
Organ support at ICU admission							
Requirement of mechanical ventilation	1494	(41.8)	603	(23.5)	698	(91.1)	<0.001
Vasopressor use	1448	(40.6)	315	(12.3)	411	(53.7)	<0.001
Renal replacement therapy	108	(3.0)	121	(4.7)	23	(3.0)	0.001
Outcome							
28-day mortality	363	(10.2)	325	(12.7)	280	(36.6)	<0.001
1-year mortality	1072	(30.0)	938	(36.6)	448	(58.5)	<0.001

Presented values are medians with interquartile ranges in parentheses or numbers with percentages in parentheses. YCC, Yonsei Cancer Center; SMC, Samsung Medical Center; MIMIC, Medical Information Mart for Intensive Care; SOFA, Sequential Organ Failure Assessment; ICU, intensive care unit; * Includes esophagus cancer, urinary cancer, gynecologic cancer, head and neck cancer, prostate cancer, pancreatic cancer, breast cancer, brain cancer, and melanoma (each cancer subtype < 5%).

**Table 2:** Performance of CanICU to predict 28-day mortality.

	YCC (*n =* 1072)		SMC (*n =* 2563)		MIMIC (*n =* 766)	
	CanICU	SOFA Score	CanICU	SOFA Score	CanICU	SOFA Score
Outcome						
28-Day Mortality	105		325		280	
1-Year Mortality	311		938		448	
Model performance						
AUC	0.939 (0.914–0.964)	0.783 (0.741–0.825)	0.775 (0.751–0.799)	0.599 (0.566–0.632)	0.753 (0.718–0.788)	0.680 (0.639–0.720)
Kappa	0.336	0.171	0.210	0.085	0.338	0.290
F1	0.841	0.728	0.723	0.743	0.688	0.753
Discrimination Indices						
Sensitivity	0.955	0.855	0.889	0.529	0.786	0.511
Specificity	0.729	0.582	0.575	0.632	0.588	0.774
PPV	0.287	0.190	0.233	0.173	0.524	0.565
NPV	0.993	0.972	0.973	0.902	0.827	0.733
Brier score	0.735	0.023	0.727	0.020	0.419	0.159

YCC, Yonsei Cancer Center; SMC. Samsung Medical Center; MIMIC. Medical Information Mart for Intensive Care; PPV. positive predictive value; NPV, negative predictive value; AUC, area under the receiver operating characteristic.

## Data Availability

The original contributions presented in the study are included in the article and supplementary material. Further inquiries can be directed to the corresponding authors.

## Supplementary Materials

The following supporting information can be downloaded at: https://www.mdpi.com/article/10.3390/cancers15030569/s1, Figure S1: Full study scheme, Figure S2: Receiver operating characteristic (ROC) curves of the CanICU model and conventional scores (SOFA and APACHE-III) for predicting 28-day mortality and one-year mortality, Figure S3: Performance of XGBoost, support vector machine, random forest, and SOFA score to predict 28-day or 1-year mortality, Figure S4: Precision-recall plots of the CanICU model and conventional scores (SOFA and APACHE-III) for predict 28-day or 1-year mortality, Figure S5: Kaplan-Meier curves according to predicted mortality by the CanICU model in each cohort, Table S1: Feature overview, Table S2: List of ICD-9/ICD-10 codes to identify each cancer, Table S3: The performance of prognostic models for 28-day mortality and one-year mortality, Table S4: The performance of CanICU to predict the 1-year mortality, Table S5. Performance according to specific sensitivities of CanICU (90%, 95% and 99%).
