# Supplementary material for: Machine Learning-Based Mortality Prediction Model for Critically Ill Cancer Patients Admitted to the Intensive Care Unit (CanICU)
Source: Cancers (Basel). 2023 Jan 17;15(3):569. doi: 10.3390/cancers15030569 (PMC9913129; doi:10.3390/cancers15030569)
Supplement: Supplementary file 1 [file cancers-15-00569-s001.zip › cancers-2054096-supplementary.pdf]

Supplementary Materials: Machine Learning-Based Mortality Prediction Model for Critically Ill Cancer Patients Admitted to the Intensive Care Unit (CanICU)

Ryoung-Eun Ko, Jaehyeong Cho, Min-Kyue Shin, Sung Woo Oh, Yeonchan Seong, Jeongseok Jeon, Kyeongman Jeon, Soonmyung Paik, Joon Seok Lim, Sang Joon Shin, Joong Bae Ahn, Jong Hyuck Park, Seng Chan You and Han Sang Kim

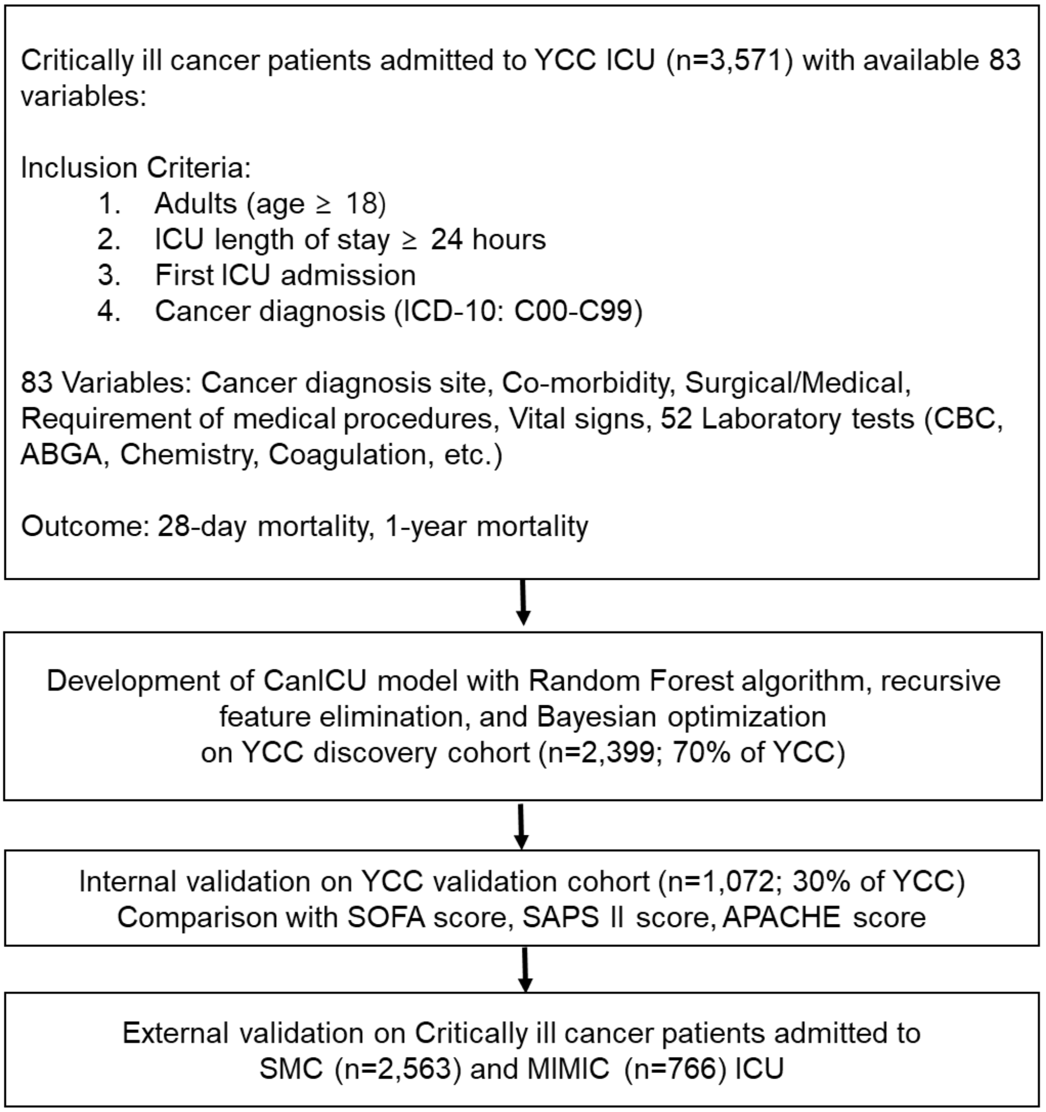

Figure S1. Full study scheme.

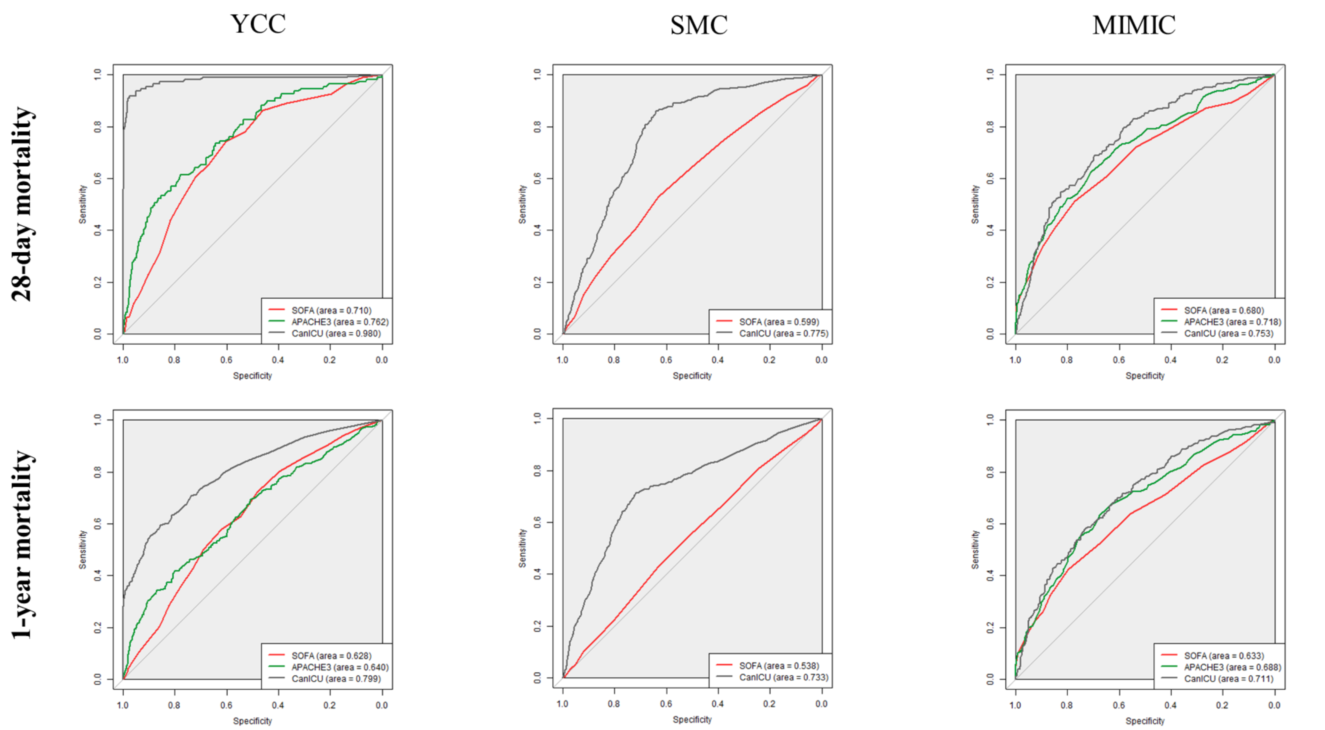

Figure S2. Receiver operating characteristic (ROC) curves of the CanICU model and conventional scores (SOFA and APACHE-III) for predicting 28-day mortality and one-year mortality. SOFA, Sequential Organ Failure Assessment; Acute Physiology and Chronic Health Evaluation, APACHE, YCC, Yonsei Cancer Center; SMC, Samsung Medical Center; MIMIC, Medical Information Mart for Intensive care.

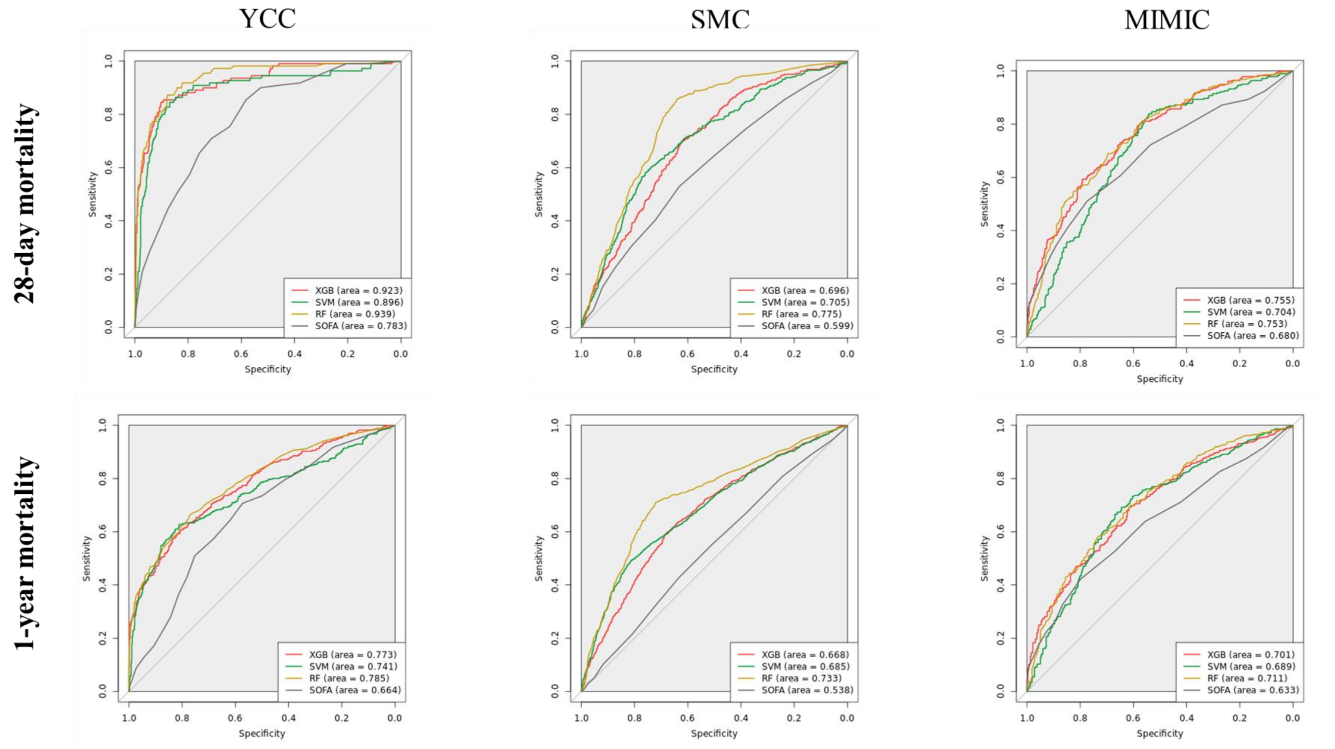

Figure S3. Performance of XGBoost, support vector machine, random forest, and SOFA score to predict 28-day or 1-year mortality. YCC, Yonsei Cancer Center; SMC, Samsung Medical Center; MIMIC, Medical Information Mart for Intensive care-III; XGB, xgboost; SVM, support vector machine; RF, random forest; SOFA, Sequential Organ Failure Assessment.

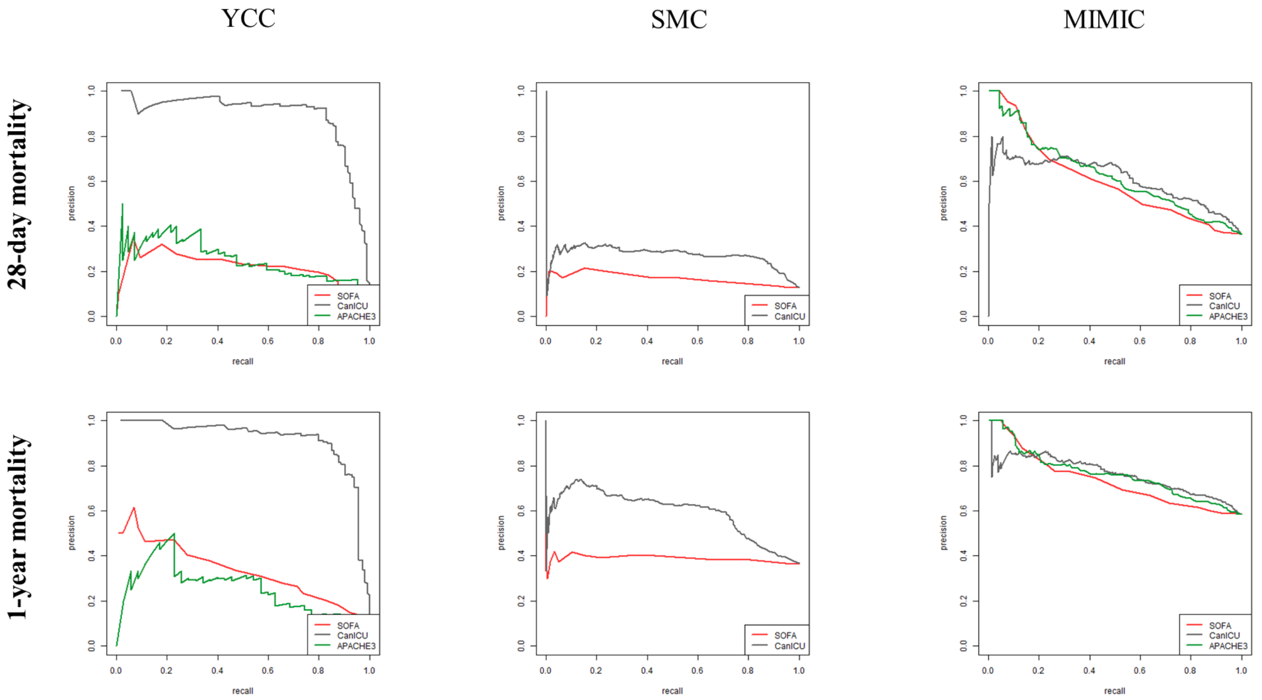

Figure S4. Precision-recall plots of the CanICU model and conventional scores (SOFA and APACHE-III) for predict 28-day or 1-year mortality. SOFA, Sequential Organ Failure Assessment; Acute Physiology and Chronic Health Evaluation, APACHE, YCC, Yonsei Cancer Center; SMC, Samsung Medical Center; MIMIC, Medical Information Mart for Intensive care.

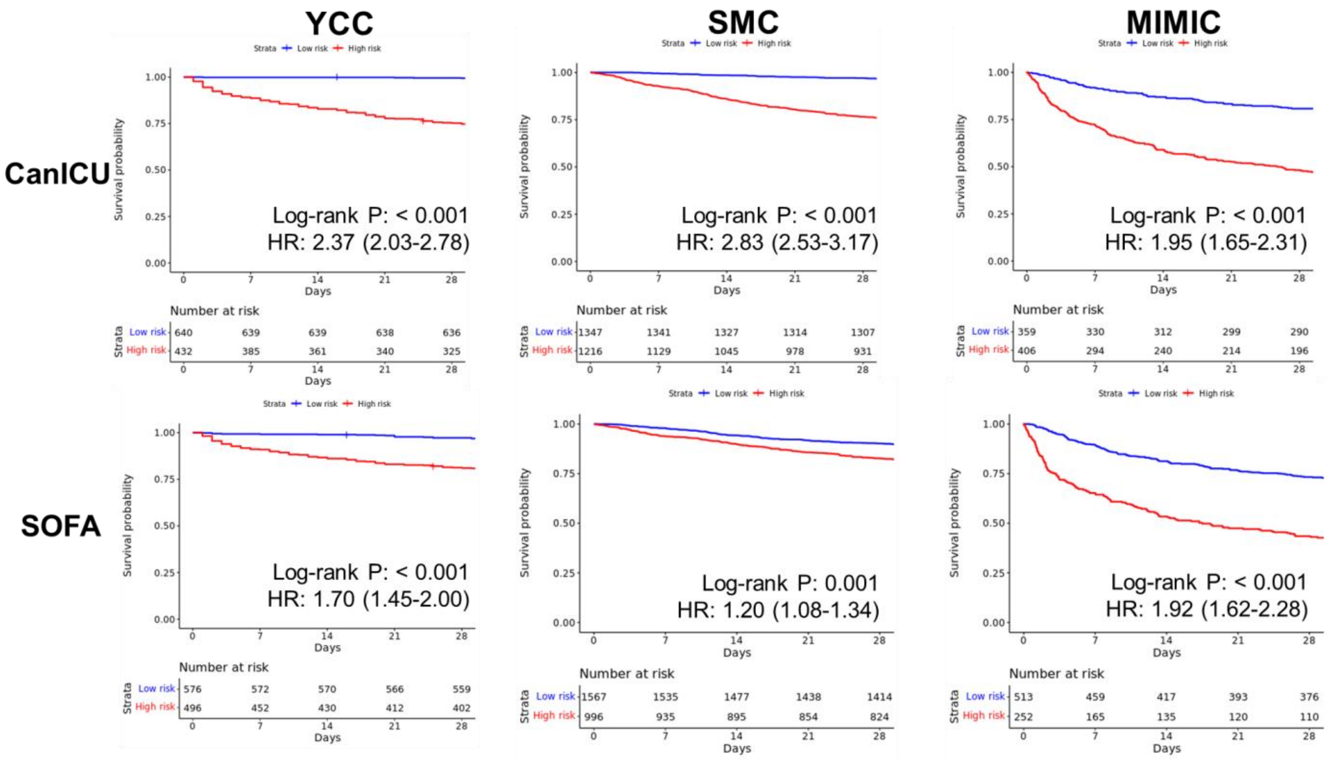

Figure S5. Kaplan-Meier curves according to predicted mortality by the CanICU model in each cohort. (**A**) 28-day survival probability of YCC validation cohort (n = 1072) (**B**) 28-day survival probability of SMC cohort (n = 2563) (**C**) 28-day survival probability of MIMIC III cohort (n = 766) (**D**) One-year survival probability of YCC validation cohort (n = 1072) (**E**) One-year survival probability of SMC cohort (n = 2563) (**F**) One-year survival probability of MIMIC-III cohort (n = 766). YCC,

Yonsei Cancer Center; SMC, Samsung Medical Center; MIMIC, Medical Information Mart for Intensive care.

Table S1. Feature overview.

Type	Category	Features
Disease information	Cancer type (14 features)	Head and neck, lung, skin and melanoma, breast, gynecology, brain, stomach, colorectal, liver, pancreas, prostate, esophagus, hematologic, urinary tract
	Comorbidity (four features)	Congestive heart failure, chronic obstructive pulmonary disease, diabetes mellitus, hypertension
Patient information	Physical characteristics (three features)	Age, sex, body weight
	Admission type (one feature)	Surgical or medical
At ICU admission information	Procedures (three features)	Mechanical ventilation, vasopressor, renal replacement therapy
	Vital signs (six features)	Heart rate, mean arterial pressure, respiratory rate, body temperature, urine output, Glasgow coma scale
	Laboratory tests (52 features)	
	-Complete blood count (20 features)	White blood cells count, lymphocyte count, neutrophil count, neutrophil-lymphocyte ratio, platelet lymphocyte ratio, platelet count, hematocrit, hemoglobin, mean corpuscular hemoglobin (MCH), mean corpuscular hemoglobin concentration (MCHC), mean corpuscular volume (MCV), mean platelet volume (MPV), platelet distribution width (PDW, %), platelet distribution width (PDW, fL), polymorphonuclear leukocytes, red blood cell count, red cell distribution width (RDW), thrombotic microangiopathy score, Delta neutrophil 1, Delta neutrophil 2
	-Arterial blood gas analysis (14 features)	PO_2/FiO_2 ratio, a/A, A, arterial alveolar oxygen tension gradient (AaDO2), base excess of blood (BE-B), base excess of extracellular fluid (BE-ECF), HCO_3-, O_2 content, pCO_2, pH, pO_2, standard bicarbonate concentration (SBC), SO_2%, TCO_2
	-Chemistry (16 features)	Albumin, alkaline phosphatase, aspartate aminotransferase (AST), alanine aminotransferase (ALT), blood urea nitrogen (BUN), calcium, cholesterol, creatinine, serum glucose, inorganic phosphate, total bilirubin, total protein, uric acid, C-reactive protein, erythrocyte sedimentation rate, lactate
	-Coagulation (two features)	Prothrombin time (international normalized ratio, INR), Activated partial thromboplastin time (aPTT)
Features selected in the final prediction model	Admission type (one feature)	Surgical or medical
	Laboratory tests (8 features)	BUN, heart rate, lactate, INR (higher is worse)<br>Albumin, PO_2/FiO_2 ratio, pH, hemoglobin (lower is worse)

Table S2. List of ICD-9/ICD-10 codes to identify each cancer.

Type	Category	Codes
ICD-9	Melanoma	172*
	Breast	174*, 175*, 239.3
	Colon	153*, 154*, 235.2
	Gyn	180*, 182*, 183*, 184*, 236.1, 236.2
	Prostate	185* 236.5
	Testes/Male GU	186*, 187.3, 187.4, 187.9, 236.4, 236.6

	Head and neck	140–149.9, 160*, 161*, 162*, 195.0
	Urinary Tract	188*, 189*, 236.7, 236.91, 239.4, 239.5
	Non-melanomatous skin cancer	173*, 238.2
	Non-colon GI	150–152.9, 155–159.9, 235*, 239.0
	Lung	162*, 235.9, 239.1
	Brain	190–192.9, 237.5, 237.6, 239.6
	Bones/soft tissue	170*, 171*, 238.1, 238.2
	Endocrine	193, 194*, 237.0, 237.4, 239.7
	Pleura/mediastinum	163*, 164*
	Non-specific site	195*, 199*, 238.8, 238.9, 239.8, 239.9
	Lymph node spread	196*
ICD-10	Malignant neoplasms of lip, oral cavity and pharynx	C00–C14
	Malignant neoplasms of digestive organs	C15–C26
	Malignant neoplasms of respiratory and intrathoracic organs	C30–C39
	Malignant neoplasms of bone and articular cartilage	C40–C41
	Melanoma and other malignant neoplasms of skin	C43–C44
	Malignant neoplasms of mesothelial and soft tissue	C45–C49
	Malignant neoplasm of breast	C50–C50
	Malignant neoplasms of female genital organs	C51–C58
	Malignant neoplasms of male genital organs	C60–C63
	Malignant neoplasms of urinary tract	C64–C68
	Malignant neoplasms of eye, brain and other parts of central nervous system	C69–C72
	Malignant neoplasms of thyroid and other endocrine glands	C73–C75
	Non-specific site	C76–C99

Table S3. The performance of prognostic models for 28-day mortality and one-year mortality.

Internal validation set: YCC (n = 1072) / outcome: Day-28 mortality (n = 105)

Characteristics	XGBoost	SVM	Random Forest	SOFA Score	APACHE-III Score
**Model Performance Metrics**					
ACC	0.910	0.788	0.752	0.610	0.799
AUC	0.923	0.896	0.939	0.783	0.784
Kappa	0.590	0.373	0.336	0.171	0.245
F1	0.949	0.868	0.841	0.729	0.882
**Discrimination Indices**					
Sensitivity	0.773	0.909	0.955	0.855	0.679
Specificity	0.926	0.774	0.729	0.582	0.808
PPV	0.545	0.315	0.287	0.190	0.216
NPV	0.973	0.987	0.993	0.972	0.970
Brier score	0.084	0.736	0.735	0.023	0.012

External validation set: MIMIC (n = 766) / outcome: Day-28 mortality (n = 280)

Characteristics	XGBoost	SVM	Random Forest	SOFA Score	APACHE-III Score
**Model Performance Metrics**					
ACC	0.696	0.657	0.661	0.678	0.679
AUC	0.755	0.704	0.753	0.680	0.718
Kappa	0.361	0.306	0.338	0.290	0.326
F1	0.751	0.703	0.688	0.753	0.737
**Discrimination Indices**					
Sensitivity	0.646	0.686	0.786	0.511	0.625
Specificity	0.724	0.640	0.588	0.774	0.710
PPV	0.575	0.523	0.524	0.565	0.554
NPV	0.780	0.779	0.827	0.733	0.767
Brier score	0.287	0.467	0.419	0.159	0.170

External validation set: SMC (n = 2563) / outcome: Day-28 mortality (n = 325)

Characteristics	XGBoost	SVM	Random Forest	SOFA Score	APACHE-III Score
**Model Performance Metrics**					
ACC	0.826	0.731	0.615	0.619	not available
AUC	0.696	0.705	0.775	0.599	not available
Kappa	0.141	0.217	0.210	0.085	not available
F1	0.902	0.830	0.723	0.743	not available
**Discrimination Indices**					
Sensitivity	0.215	0.585	0.889	0.529	not available
Specificity	0.914	0.752	0.575	0.632	not available
PPV	0.267	0.255	0.233	0.173	not available
NPV	0.889	0.926	0.973	0.902	not available
Brier score	0.029	0.759	0.727	0.020	not available

Internal validation set: YCC (n = 1072) / outcome: One-year mortality (n = 311)

Characteristics	XGBoost	SVM	Random Forest	SOFA Score	APACHE-III Score
**Model Performance Metrics**					
ACC	0.765	0.766	0.774	0.616	0.673
AUC	0.773	0.741	0.785	0.664	0.590
Kappa	0.350	0.401	0.420	0.240	0.174
F1	0.849	0.842	0.848	0.670	0.775
**Discrimination Indices**					
Sensitivity	0.320	0.446	0.452	0.707	0.424
Specificity	0.973	0.915	0.925	0.573	0.758
PPV	0.845	0.710	0.737	0.436	0.375
NPV	0.754	0.780	0.783	0.807	0.793
Brier score	0.084	0.736	0.735	0.116	0.072

External validation set: MIMIC (n = 766) / outcome: One-year mortality (n = 448)

Characteristics	XGBoost	SVM	Random Forest	SOFA Score	APACHE-III Score
**Model Performance Metrics**					
ACC	0.594	0.569	0.581	0.578	0.629
AUC	0.701	0.689	0.711	0.633	0.688
Kappa	0.236	0.190	0.224	0.203	0.280
F1	0.631	0.609	0.636	0.611	0.630
**Discrimination Indices**					

Sensitivity	0.422	0.400	0.368	0.422	0.536
Specificity	0.836	0.808	0.881	0.799	0.761
PPV	0.784	0.746	0.813	0.747	0.759
NPV	0.507	0.489	0.497	0.495	0.538
Brier score	0.287	0.467	0.419	0.372	0.170
External validation set: SMC (*n* = 2563) / outcome: One-year mortality (*n* = 938)					
**Characteristics**	**XGBoost**	**SVM**	**Random Forest**	**SOFA Score**	**APACHE-III Score**
**Model Performance Metrics**					
ACC	0.651	0.686	0.690	0.525	not available
AUC	0.668	0.685	0.733	0.538	not available
Kappa	0.083	0.233	0.260	0.056	not available
F1	0.779	0.787	0.785	0.576	not available
**Discrimination Indices**					
Sensitivity	0.099	0.292	0.344	0.551	not available
Specificity	0.969	0.913	0.890	0.510	not available
PPV	0.650	0.660	0.643	0.393	not available
NPV	0.651	0.691	0.702	0.663	not available
Brier score	0.029	0.759	0.727	0.134	not available

**Model**	**XGBoost**	**SVM**	**Random Forest**	**SOFA Score**	**APACHE-III Score**
	AUC (95% CI)				
YCC,	0.923	0.896	0.939	0.783	0.784
28-day	0.893–0.952	0.859–0.934	0.914–0.964	0.741–0.825	0.700–0.868
MIMIC,	0.755	0.704	0.753	0.680	0.718
28-day	0.719–0.790	0.667–0.742	0.718–0.788	0.639–0.720	0.679–0.756
SMC,	0.696	0.705	0.775	0.599	not available
28-day	0.667–0.724	0.675–0.735	0.751–0.799	0.566–0.632	not available
YCC,	0.773	0.741	0.785	0.664	0.640
one-year	0.741–0.804	0.706–0.775	0.755–0.816	0.629–0.698	0.603–0.676
MIMIC,	0.701	0.689	0.711	0.633	0.688
one-year	0.664–0.738	0.651–0.728	0.674–0.747	0.594–0.672	0.650–0.725
SMC,	0.668	0.685	0.733	0.538	not available
one-year	0.646–0.689	0.663–0.707	0.712–0.753	0.515–0.561	not available

YCC, Yonsei Cancer Center; SMC, Samsung Medical Center; MIMIC, Medical Information Mart for Intensive care; XGBoost, extreme gradient boosting; SVM, support vector machine; SOFA, Sequential Organ Failure Assessment; APACHE, Acute Physiology and Chronic Health Evaluation; ACC, accuracy; AUC, area under the receiver operating characteristic; PPV, positive predictive value; NPV, negative predictive value.

Table S4. The performance of CanICU to predict the 1-year mortality.

	**YCC (*n* = 1072)**		**SMC (*n* = 2563)**		**MIMIC (*n* = 766)**	
	**CanICU**	**SOFA Score**	**CanICU**	**SOFA Score**	**CanICU**	**SOFA Score**
**Model Performance**						
ACC	0.774	0.616	0.690	0.525	0.581	0.578
AUC	0.785 (0.755–0.816)	0.664 (0.629–0.698)	0.733 (0.712–0.753)	0.538 (0.515–0.561)	0.711 (0.674–0.747)	0.633 (0.594–0.672)
Kappa	0.420	0.240	0.260	0.056	0.224	0.203
F1	0.848	0.670	0.785	0.576	0.636	0.611
**Discrimination Indices**						
Sensitivity	0.452	0.707	0.344	0.551	0.368	0.422

Specificity	0.925	0.573	0.890	0.510	0.881	0.799
PPV	0.737	0.436	0.643	0.393	0.813	0.747
NPV	0.783	0.807	0.702	0.663	0.497	0.495
Brier score	0.735	0.116	0.727	0.134	0.419	0.372

YCC, Yonsei Cancer Center; SMC, Samsung Medical Center; MIMIC, Medical Information Mart for Intensive care; ACC, accuracy; AUC, area under the receiver operating characteristic; PPV, positive predictive value; NPV, negative predictive value.

Table S5. Performance according to specific sensitivities of CanICU (90%, 95% and 99%).

**Model**	**Sensitivity 90%**			**Sensitivity 95%**			**Sensitivity 99%**		
	**Number of Patients Recommended for ICU Admission**	**PPV**	**NPV**	**Number of Patients Recommended for ICU Admission**	**PPV**	**NPV**	**Number of Patients Recommended for ICU Admission**	**PPV**	**NPV**
YCC, 28-day	116/1072 (10.82%)	0.784	0.990	173/1072 (16.14%)	0.555	0.994	325/1072 (30.32%)	0.308	0.999
MIMIC, 28-day	554/766 (72.32%)	0.455	0.868	631/766 (82.38%)	0.422	0.896	737/766 (96.21%)	0.376	0.897
SMC, 28-day	1392/2564 (54.29%)	0.210	0.973	1819/2564 (70.94%)	0.170	0.979	2396/2564 (93.45%)	0.134	0.982
YCC, one-year	731/1072 (68.19%)	0.394	0.889	808/1072 (75.37%)	0.376	0.917	1072/1072 (100%)	0.304	not available
MIMIC, one-year	619/766 (80.81%)	0.653	0.701	676/766 (88.25%)	0.629	0.744	761/766 (99.35%)	0.583	0.200
SMC, one-year	2054/2564 (80.11%)	0.412	0.822	2239/2564 (87.32%)	0.396	0.843	2564/2564 (100%)	0.366	not available

YCC, Yonsei Cancer Center; SMC, Samsung Medical Center; MIMIC, Medical Information Mart for Intensive care; PPV, positive predictive value; NPV, negative predictive value.
